# The Golden Beauty: Brain Response to Classical and Renaissance Sculptures

**DOI:** 10.1371/journal.pone.0001201

**Published:** 2007-11-21

**Authors:** Cinzia Di Dio, Emiliano Macaluso, Giacomo Rizzolatti

**Affiliations:** 1 Dipartimento di Neuroscienze, Università di Parma, Parma, Italy; 2 Fondazione Santa Lucia, Neuroimaging Laboratory, Rome, Italy; University of Minnesota, United States of America

## Abstract

Is there an objective, biological basis for the experience of beauty in art? Or is aesthetic experience entirely subjective? Using fMRI technique, we addressed this question by presenting viewers, naïve to art criticism, with images of masterpieces of Classical and Renaissance sculpture. Employing proportion as the independent variable, we produced two sets of stimuli: one composed of images of original sculptures; the other of a modified version of the same images. The stimuli were presented in three conditions: observation, aesthetic judgment, and proportion judgment. In the observation condition, the viewers were required to observe the images with the same mind-set as if they were in a museum. In the other two conditions they were required to give an aesthetic or proportion judgment on the same images. Two types of analyses were carried out: one which contrasted brain response to the canonical and the modified sculptures, and one which contrasted beautiful *vs*. ugly sculptures as judged by each volunteer. The most striking result was that the observation of original sculptures, relative to the modified ones, produced activation of the right insula as well as of some lateral and medial cortical areas (lateral occipital gyrus, precuneus and prefrontal areas). The activation of the insula was particularly strong during the observation condition. Most interestingly, when volunteers were required to give an overt aesthetic judgment, the images judged as beautiful selectively activated the right amygdala, relative to those judged as ugly. We conclude that, in observers naïve to art criticism, the sense of beauty is mediated by two non-mutually exclusive processes: one based on a joint activation of sets of cortical neurons, triggered by parameters intrinsic to the stimuli, *and* the insula (objective beauty); the other based on the activation of the amygdala, driven by one's own emotional experiences (subjective beauty).

## Introduction

One of the most debated issues in aesthetics is whether beauty may be defined by some objective parameters or whether it merely depends on subjective factors. The first perspective goes back to Plato's *objectivist view* of aesthetic perception, in which beauty is regarded as a property of an object that produces a pleasurable experience in any suitable viewer. This stance may be rephrased in biological terms by stating that human beings are endowed with species-specific mechanisms that resonate in response to certain parameters present in works of art. The alternative stance is that the viewers' evaluation of art is fully *subjective.* It is determined by experience and personal values (see [1], [2]).

Although it is commonly accepted that subjective criteria play a major role in one's aesthetic experience (see [3]), it is also reasonable to accept that there exist specific biologically-based principles which may facilitate the perception of beauty in the beholder. After all, new artists typically first master the ability to represent standard principles of beauty, such as symmetry and proportion, and only then eventually bend these rules to represent their overall vision of the world (see [4]).

**Figure 1:**
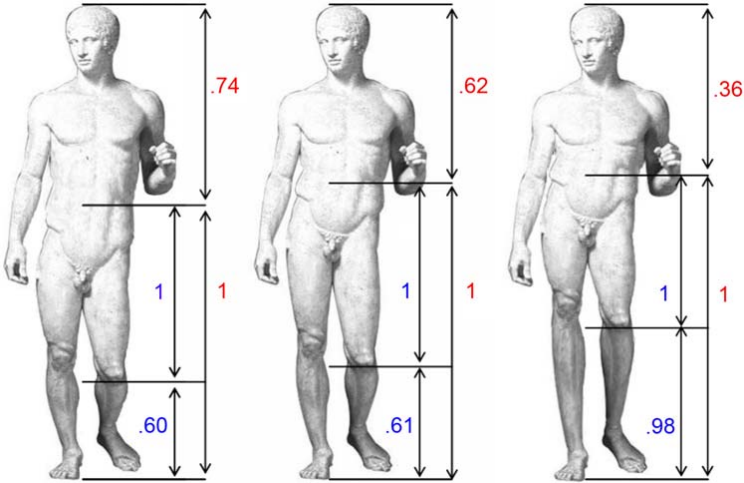
Example of canonical and modified stimuli. The original image (Doryphoros by Polykleitos) is shown at the centre of the figure. This sculpture obeys to canonical proportion (golden ratio = 1∶1.618). Two modified versions of the same sculpture are presented on its left and right sides. The left image was modified by creating a short legs∶long trunk relation (ratio = 1∶0.74); the right image by creating the opposite relation pattern (ratio = 1∶0.36). All images were used in behavioral testing. The central image (judged-as-beautiful on 100%) and left one (judged-as-ugly on 64%) were employed in the fMRI study.

In the present study we investigated the aesthetic effect of objective parameters in the works of art by studying brain activations (fMRI) in viewers naïve to art criticism who observed images of sculptures selected from masterpieces of Classical and Renaissance art that are commonly accepted as normative Western representations of beauty. An important feature that characterized the present study distinguishing it from others that also have attempted to clarify the neural correlates of aesthetic perception [5]–[8] was the use of two sets of stimuli that were identical in every aspects but one: proportion. More specifically, a parameter that is considered to represent the ideal beauty, namely the golden ratio (1:0.618; for reviews see [9], [10]), was modified to create a degraded aesthetic value of the same stimuli in a controlled fashion (Figure 1). Stimulus manipulation was very contained and in no cases were the modified sculptures judged as deformed representations of the human body, as assessed in post-scanning debriefing. Another important feature of the present study was that the same stimuli were presented in experimental conditions that varied in the instructions given to the participants. In one condition-observation (O)–viewers were asked to observe the sculptures as if they were in a museum, without any explicit request to judge them. By inducing a “simply enjoy” contextual frame and without having the volunteers perform any specific cognitive task, we meant to elicit a most spontaneous/unbiased brain response to the artworks. In a second-aesthetic judgment (AJ)- and third -proportion judgment (PJ)- condition, on the other hand, the viewers had to judge the stimuli on the basis of their aesthetic or proportion quality, respectively. Therefore, in both these conditions the participants were involved in an additional cognitive evaluation of the stimuli. Whereas the aesthetic judgment condition allowed us to determine brain activations in response to the volunteer's subjective evaluation of the stimuli, the PJ condition was used to observe brain response during a task of overt proportion evaluation.

In order to assess both “objective” and “subjective” aesthetic values, two types of analysis were carried out. In the first one, aimed at establishing the neural responses to objective beauty parameters, we contrasted brain activations during the presentation of the canonical sculptures *vs*. their modified counterparts. The underlying rationale was that the canonical proportions intrinsic to the original works of art would elicit enhanced activity in areas mediating pleasure and, in particular, in the insula, the cortical region known to be involved in the *feeling* of emotion (see [11]–[15]). We also expected signal increase to be particularly strong during the observation condition, where brain response to the artworks was not interfered with by additional cognitive requests (i.e. aesthetic or proportion judgment). The second type of analysis, on the other hand, was aimed at the evaluation of brain responses related to the overt subjective appreciation of the stimuli by contrasting the brain activations obtained during the presentation of the judged-as-beautiful against the judged-as-ugly images. In this analysis, we expected the judged-as-beautiful images to produce a stronger activation, than the judged-as-ugly images, in areas involved in the subjective emotional appraisal of the stimuli. In this case, however, we did not bring forward any specific prediction due to the divergent existing evidence in the field.

## Materials and Methods

### Participants

Fourteen healthy right-handed volunteers (8 males, 6 females; mean age 24.5, range 12 years) participated in this study. They were educated undergraduate or graduate students, with no experience in art theory. After receiving an explanation of the experimental procedure, participants gave their written informed consent. The study was approved by the independent Ethics Committee of the Santa Lucia Foundation (Scientific Institute for Research Hospitalization and Health Care).

### Stimuli

Fifteen 2-dimentional images of Classical and Renaissance sculptures were chosen following the selection method described in Supporting Information (Text S1). All the original pictures met the criteria of canonical proportions defined by the ratio 1:1.618 between body parts; among the 15 modified image-versions, 7 presented a ‘long-trunk, short-legs’ modification (range = 1∶1.47-1∶1.59), whereas the remaining 8 images presented the opposite pattern of modification (range = 1∶1.64-1∶1.82). Twenty sculptures represented male bodies and 10 female bodies.

### Paradigm

The stimuli were presented in three experimental conditions: observation (O), aesthetic judgment (AJ), and proportion judgment (PJ). Each participant underwent 6 separate fMRI runs, repeating each condition twice. The condition order was maintained fixed across all participants, with observation condition first, explicit aesthetic judgment second, and explicit proportion judgment, last. By keeping the observation runs first, we aimed at measuring unbiased (spontaneous) brain responses to the type of the stimuli (canonical and modified). To make sure that volunteers were not biased in their aesthetic judgment by explicit proportion evaluation, the aesthetic judgment condition always preceded the proportion judgment runs.

Within each run we presented 30 stimuli (15 canonical and 15 modified) in a randomized order, but never repeating the same image within a run. A question mark instructed the participants to respond to the images after a 4s-fix interval following each stimulus presentation by using a response box placed inside the scanner.

### Task

Participants lay in the scanner in a dimly lit environment. The stimuli were presented on a black background and were displayed on a screen visible through a mirror mounted on the interior of the head coil. At the beginning of each session, a 5 s visual instruction informed the volunteers about the upcoming condition/task. On each trial, a 400ms central fixation point plus 1000 ms blank-screen interval preceded the presentation of the sculpture stimulus. The stimulus then appeared at the centre of the screen for 2 s (see also [5], [16]) and it was followed by another 4 s blank-screen interval. After this, a question mark instructed the observer to respond to the stimulus (see below). The question mark remained on screen for 400 ms and was followed by a jittered interval ranging 2–5 s, with a uniform distribution.

During observation condition (O), the volunteers were required to observe the images as if they were in a museum and, when the question mark appeared, they had to indicate whether they paid attention to the picture or not. During the aesthetic and proportion judgment conditions, the volunteers were required to decide whether they liked the image (AJ) or whether they found it proportional (PJ), respectively. Thus, all 3 conditions required a response from the participants. Using the index or middle finger of the right hand, the participants answered yes or no, according to the instruction presented at the start of each run. Specifically, before the observation sessions, the participants were instructed to answer ‘yes’ if they paid attention to the stimulus just presented, whereas to press ‘no’ to indicate that they did not pay attention to the stimulus. The question ‘did you pay attention to the image?’ was introduced to make sure that participants were actually looking at the stimuli during fMRI scanning. During AJ condition, participants were required to indicate ‘yes’ if they aesthetically liked the image and ‘no’ if they did not. Finally, PJ condition required the observers to explicitly indicate whether they thought that the image was proportional by pressing ‘yes’ or if they thought that the image was disproportionate by pressing ‘no’.

The volunteers underwent six subsequent scanning runs, each lasting approximately 5.6 min. Each fMRI runs consisted of 30 trials with each sculpture images presented once.

### Image acquisition

Functional images were acquired with a Magnetom Vision MRI scanner (Siemens, Erlangen, Germany) operating at 3T. Blood oxygenation level dependent (BOLD) contrast was obtained using echo-planar T2* weighted imaging (EPI). The acquisition of 32 transverse slices with an effective repetition time of 2.08 s, provided coverage of the whole cerebral cortex. The in-plane resolution was 3×3 mm.

### Data analysis

Two types of analyses of fMRI data were performed. A stimulus-based analysis (‘objective beauty’) considered only the type of image that was presented to the participants: i.e. with canonical (C) or modified (M) proportions. The second analysis (‘subjective beauty’) categorized each sculpture image according to the behavioral responses measured during AJ runs. For this analysis, we included only images that were consistently judged either beautiful (B) or ugly (U) in both runs requiring aesthetic judgment.

Event-related fMRI data were processed with SPM2 (http://www.fil.ion.ucl.ac.uk). The first four image volumes of each run were discarded to allow for stabilization of longitudinal magnetization. For each participant, the remaining 162 volumes were realigned with the first volume, and the acquisition timing was corrected using the middle slice as reference [17]. To allow inter-subject analysis, images were normalised to the Montreal Neurological Institute (MNI) standard space [18], using the mean of the 162 functional images. All images were smoothed using an isotropic Gaussian kernel (full width at half maximum = 10 mm).

Statistical inference was based on a random effects approach [19]. This comprised two steps. First, for each subject, the data were best-fitted (least-square fit) at every voxel using a linear combination of the effects of interest. The effects of interest were the timing of the fixation point onsets, the presentation times of the sculptures (C & M; or B & U), and the presentation times of the question mark that cued overt responses. All event-types were convolved with the SPM2 standard haemodynamic response function (HRF). Linear compounds (contrasts) were used to determine common effect (C+M *vs.* rest) and differential effects associated with the presentation of the sculptures (C-M and M-C; or B-U and U-B), separately for each of the three conditions (O, AJ and PJ). For each subject, this led to the creation of six contrast-images, that is three contrasts C+M *vs*. rest–one for each condition, and three contrasts C-M *vs*. rest, again one for each condition. Additionally, three contrast-images were also created, which contrasted judged-as-beautiful *vs*. judged-as-ugly images for each condition.

These contrast-images then underwent the second step that comprised three separate ANOVAs. One considering overall pattern of activation ‘C+M *vs.* rest’ modeled for each condition; one considering ‘objective beauty’ (C *vs*. M) modeled for each condition; and one considering ‘subjective beauty’ (B *vs*. U) for each condition. Finally, for each of the three separate ANOVAs, linear compounds were used to compare these effects, now using between-subjects variance. Correction for non-sphericity [20] was used to account for possible differences in error variance across conditions and any non-independent error terms for the repeated measures.

The following contrasts were tested. First, within the “common effects”, ANOVA (C+M *vs*. rest) averaging across all experimental conditions (O, AJ, PJ). For this, the SPM-maps were thresholded at *P*-corrected = 0.05 (voxel-level). The other two ANOVAs assessed any stimulus -specific effect (‘objective’: C-M, M-C; or ‘subjective’: B-U, U-B). We tested for main effects of stimulus across the three experimental conditions (O, AJ, PJ); and for interactions between stimulus and condition. Additional contrasts explored simple effects separately for the different conditions (e.g. B-U, during AJ only). For all these stimulus-specific effects, we used *P*-corrected = 0.05 at the cluster-level (cluster size estimated with a voxel-level threshold of *P*-uncorrected = 0.001, extent threshold = 10 voxels).

In addition, because of our prior hypothesis concerning the possible involvement of the insula in aesthetic appreciation, we used a small volume correction procedure [21] to test for the effect of ‘objective beauty’ (C-M; within and across O/AJ/PJ conditions) specifically in this region. The search volume was derived from [10] (see also [14]–[15]) centering a sphere at MNI x, y, z = 30, 18, 18; with a radius of 10 mm.

## Results and Discussion

### fMRI behavioral data

The viewers' evaluation of the stimuli, as expressed in the aesthetic judgment condition, showed that the canonical images were mostly evaluated positively (76%, sd = 0.18), whereas the modified images were generally scored with a negative rating (63%, sd = 0.25). This finding was in accord with a preliminary behavioral testing used for images selection that also showed the relevance of proportion in aesthetic evaluation. In this test violation of canonical proportions accounted for 77% of the variance in aesthetic rating (partial Eta^2^; see Supporting Information Text S1 for details on the preliminary behavioral experiment).

### Overall effect of viewing the sculptures

MRI analysis was carried out by first assessing the overall effect of viewing the sculptures contrasting canonical (C) and modified (M) images (pooled together, C+M) with rest, across all three conditions (O, AJ, PJ; *P*-corrected<0.05).

**Figure 2:**
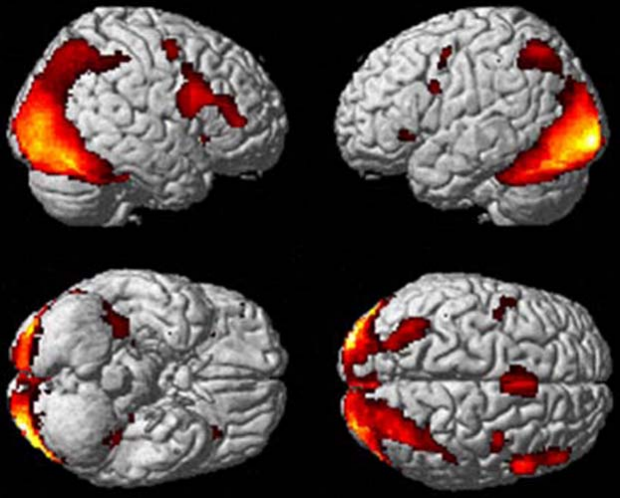
Brain activation of canonical and modified sculptures *vs*. rest. The analysis was carried out by averaging activity across the three experimental conditions (observation, aesthetic judgment, proportion judgment). Group-averaged statistical parametric maps are rendered onto the MNI brain template (*P*-corrected<0.05).

As shown in Figure 2, activations were found in occipital and temporal visual areas, including lingual and fusiform gyri. Additionally, activations were observed in the inferior parietal lobule (IPL) bilaterally, in the SMA/pre-SMA complex, ventral premotor areas, and in the posterior part of right inferior frontal gyrus (IFG). Signal increase was also found in the insula and hippocampus. Most of the activations were bilateral, although stronger in the right hemisphere. These results are summarized in Table 1.

**Table 1:** Brain activity reflecting the common effects of Canonical and Modified images *vs*. baseline across conditions (observation; aesthetic judgment; proportion judgment).

Brain structure		Sphere	Maxima x y z	Z	p. corr (vx)
Occipital Lobe					
	Inferior occipital gyrus (LO)	L	−45 −84 −6	Inf	0.000
		R	38 −88 −10	Inf	0.000
	Middle occipital gyrus	L	−32 −96 −6	Inf	0.000
		R	28 −92 0	Inf	0.000
		R	30 −92 −2	Inf	0.000
		R	50 −78 −8	7.82	0.000
Parietal Lobe					
	Supramarginal gyrus	R	64 −20 38	5.08	0.006
Frontal Lobe					
	Middle frontal gyrus	R	38 0 54	5.49	0.001
		R	38 −2 54	4.89	0.015
		R	38 0 52	4.65	0.041
	Inferior frontal gyrus	R	50 14 24	7.32	0.000
		R	52 14 24	5.36	0.002
		R	45 40 8	5.33	0.002
		R	50 34 18	5.25	0.003
		R	48 35 14	5.24	0.003
	Precentral gyrus	R	50 10 12	6.21	0.005
		R	54 8 42	4.84	0.019
	Precentral gyrus	L	−56 2 42	4.58	0.036
		L	−50 8 30	4.62	0.047
		L	−52 6 26	4.56	0.05
	Supplementary motor area	-	0 10 52	6.36	0.000
	Supplementary motor area	R	4 16 48	5.16	0.005
		R	2 16 50	6.01	0.000
		R	14 8 58	5.21	0.004
Subcortical/insula					
	Ippocampus	R	24 −32 −6	5.49	0.001
		R	22 −32 −6	5.35	0.002
	Ippocampus	L	−22 −32 −6	6.14	0.000
				4.92	0.013
	Insula	R	36 20 −6	5.05	0.008
	Insula	L	−34 24 −4	5.58	0.001
Cerebellum					
	Cerebellum 4-5	R	32 −34 −28	4.81	0.021
					

Among the visual activations, besides the primary visual cortex, signal increase was found in the lateral occipital cortex and the inferior temporal lobe (shape sensitive areas), as well as in the MT/MST complex. This last finding, although surprising at first considering that the MT/MST complex is involved in the analysis of motion [22]–[24], is consistent with previous data showing that activation of these areas may be elicited by static images that imply motion [25]. Most noteworthy was the activation of the inferior parietal lobule and especially of the premotor cortex. These areas are known to become active during the observation of actions done by others (see [26]). It is likely that their activation was dependent on the intrinsic dynamic properties of the sculptures used in this study and the sense of action that they evoked in the observer (see [27]).

### Canonical *vs.* Modified Sculptures: “Objective Beauty”

**Figure 3:**
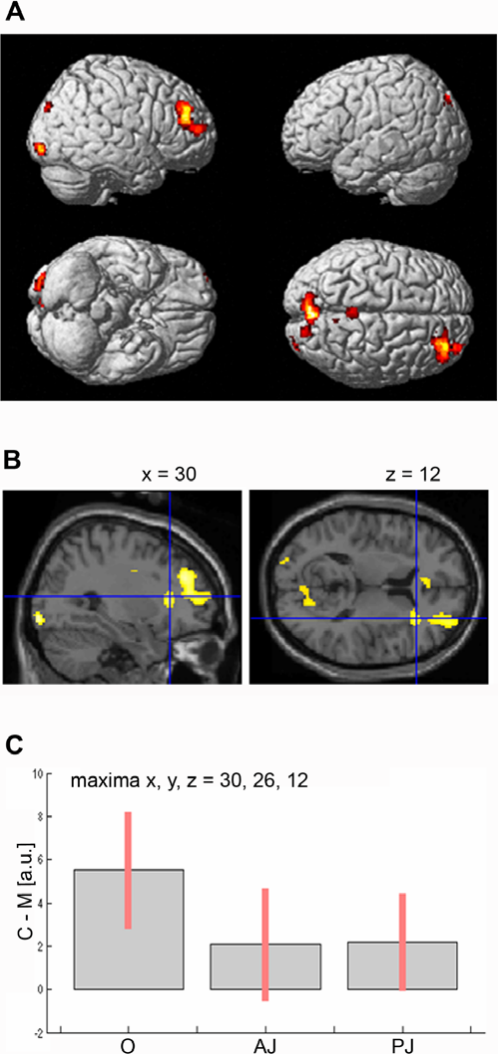
Brain activation in the contrast canonical *vs*. modified stimuli. a, Main effect of canonical *vs*. modified sculptures across conditions rendered onto the MNI brain template. b, Parasagittal and coronal view showing activations of the right insular region in the main effect. c, Activity profile of the right insula. For each condition (O, AJ, PJ) the signal plots show the difference between canonical (C) minus modified (M) sculptures in arbitrary units (a.u), +/− 10% confidence intervals (*P*-corrected<0.05).

The direct contrast of canonical *vs*. modified images across the three experimental conditions revealed signal increase for the canonical stimuli in the right occipital cortex extending into lingual gyrus; in the precuneus bilaterally; in the right posterior cingulate gyrus; and in the depth of right inferior frontal sulcus extending to the adjacent convexity of the middle frontal gyrus (*P*-corrected<0.05; Figure 3a; see also Table 2a).

**Table 2:** Brain activity reflecting the main effect (a) and the simple effect (b) of Canonical *vs.* Modified images.

Brain structure	Sphere	Maxima x y z	Z	p. corr Cluster level
**a Main effect (C-M)**				
Medial parietal lobe/Precuneus	R	12, −52, 46	3.79	0.04
	L	−2, −42, 58	3.21	
Posterior cingulum	R	8, −52, 30	3.33	
Inferior occipital gyrus	R	30, −94, −8	3.75	0.0001
Lingual gyrus	R	16, −66, −6	3.56	
Cuneus	L	−4, −78, 30	3.55	
Inferior frontal gyrus	R	44, 42, 20	3.65	0.03
Middle frontal gyrus	R	30, 40, 30	3.65	
**b Simple effect Observation (C-M)**				
Anterior insula/frontal operculum	R	36, 22, 16	3.86	0.016
Middle frontal gyrus	R	38, 36, 20	3.62	
Superior frontal gyrus	R	18, 44, 26	3.31	

The lateral occipital cortex (LOC, [28], [29]) and the temporal visual areas are known to be responsive to the presentation of body parts or even the whole human body [30], [31]. Signal increase within these areas may be therefore due to a greater representation of canonical body structures relative to the disproportionate ones. The activation of the medial parietal areas and of the prefrontal lobe, on the other hand, might be related to mnemonic functions (e.g. [32], [33]; for review see [34]), possibly elicited by the retrieval of plausible motor configurations, better represented by the proportional material.

The central hypothesis underlying the present study was that the contrast of canonical *vs*. modified stimuli would produce signal enhancement within the insula. Accordingly, we carried out a small volume correction within the main effects analysis (C-M) using the anatomical coordinates reported in [12] on the feeling of emotion (see also [14], [15]). The results revealed a significant signal increase in the *anterior sector* of the right insular cortex extending to the operculum region (maxima x, y, z = 30, 26, 12; Figure 3b, *P*<0.05, corrected for small volume).

This effect was particularly strong during *observation* condition (*P*<0.02, corrected for the whole brain volume, Table 2b; *P* = 0.005, corrected for small volume), that is in the condition in which the volunteers were in a merely observational (museum-like) context (see Figure 3c). Signal increase in AJ and PJ conditions, on the other hand, was virtually the same. The most likely interpretation for this result stands in the different cognitive demands between the first (O) and the last two (AJ, PJ) conditions. In the latter, in fact, the explicit request of overtly judging the stimuli diverted the volunteers' attention resources towards a specific cognitive demand, thus lessening the natural neural response within the insula.

These data are in apparent contrast with some previous findings where symmetry was employed as an objective parameter of aesthetic evaluation [8]. In this study, the authors *did* find significant activation in the anterior insula in the comparison of aesthetic judgment *vs*. control condition as well as in symmetry judgment *vs*. control condition. However, they considered those areas that were activated by both aesthetic and symmetry judgment to be not involved in pure aesthetic judgment and hence omitted them from the analysis that directly contrasted brain activity for the judged-as-beautiful *vs*. the judged-as-ugly stimuli. In this way, therefore, they also disregarded the insular activation elicited by objective parameters (i.e. symmetry) intrinsic to the stimuli and involved in mediating the sense of beauty.

The question now arises of what possible mechanisms are responsible for the insula activation during the observation of canonical sculptures. The anterior sector of the insula has an agranular/disgranular cytoarchitectonic organization and is characterized by extensive connections with limbic structures and with centers involved in autonomic functions [35]–[37]. Functionally, anterior insula is thought to mediate feelings associated with specific emotional states [38], [11]–[15]. Now, considering the pattern of activity described in the main effect (C+M *vs.* rest), there are two concurrent possibilities that may explain insula activation. One is that in LOC and in the parietal cortex there are neurons *specifically* sensitive to the canonic body images and that have privileged access to the insula. Alternatively, one may suppose that the canonical sculptures simply determined a *stronger* activation of cortical neurons sending their output to the insula.

Another possible explanation, based on both main and simple effect analyses (C-M), is that the insula was activated, not by simplest aspects of the visual stimuli (e.g. shape or motion), but rather by higher order information coming from prefrontal areas 45 and 46. Studies in primates [39] showed that area 45 integrates information about object shape with that about actions. While human left area 45 subserves language functions, it is plausible that human right area 45, selectively activated in the present experiment, could be involved in action/shape integration as well. In this light, canonical stimuli could be more efficiently coded in this area and determined, therefore, a stronger activation of the insula relative to the modified one. In this context, also the functional role of prefrontal area 46 could be noteworthy in confronting information from memory (e.g. standard body configuration) with online incoming information (observation of canonical and modified stimuli).

To summarize, we propose that the positive emotional *feeling* elicited in the viewer by the canonical images was determined by a preferential coding of these images, relative to the modified ones, by various cortical areas and by a concurrent, *joint* activation of the anterior insula.

### Judged-as-Beautiful *vs.* Judged-as-Ugly Sculptures: “Subjective Beauty”

With this further analysis, we investigated the neuronal substrate associated with subjective appreciation of the sculptures as expressed by each participant in the AJ condition (2 runs). Behavioral data showed that 49% and 38% of stimuli were consistently judged, respectively, beautiful (B) and ugly (U) over both AJ runs, whereas 13% was rated inconsistently. Only the stimuli that were rated in a consistent way were employed for analysis.

**Figure 4:**
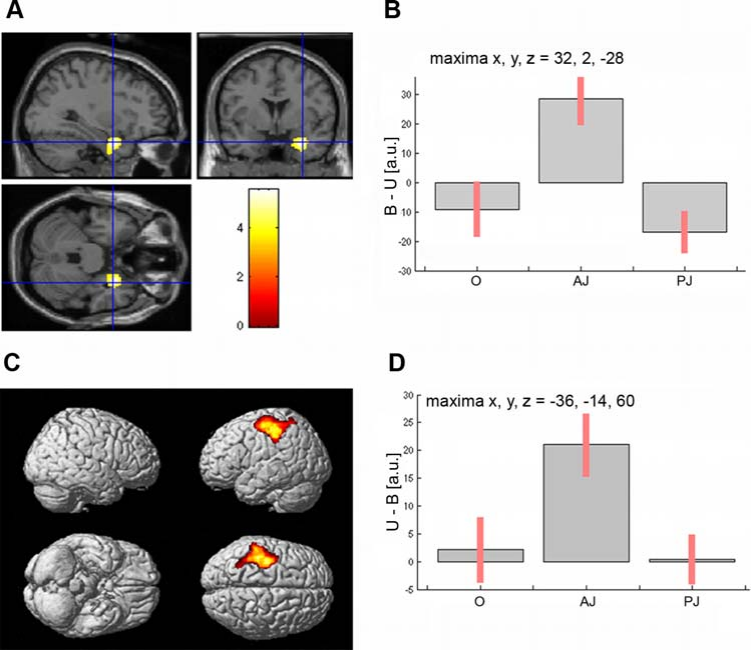
Brain activations in the contrasts “judged-as-beautiful *vs*. judged-as-ugly” and “judged-as-ugly *vs*. judged-as-beautiful” stimuli. a, Parasagittal, coronal and transaxial sections showing activation of the right amygdala in the interaction stimulus (beautiful *vs*. ugly)×condition (observation; aesthetic judgment; proportion judgment). b, Activity profile of the right amygdala. For each condition (O = observation, AJ = aesthetic judgment, PJ = proportion judgment) the signal plots show the difference between beautiful (B) minus ugly (U)-as judged sculptures in arbitrary units (a.u), +/− 10% confidence intervals. c, Statistical parametric maps rendered onto the MNI brain template showing activity within left somatomotor cortex in the contrast of ugly *vs.* beautiful stimuli averaged across the three conditions. d, Activity profile (ugly-beautiful) of the left motor cortex. For each condition (O, AJ, PJ) the signal plots show the difference between ugly (U) minus beautiful (B)-as judged sculptures in arbitrary units (a.u), +/− 10% confidence intervals (*P*-corrected<0.05).

The judged-as-beautiful images selectively activated the right amygdala. This effect was observed for the aesthetic judgment condition, as demonstrated by the stimulus×condition interaction analysis (maxima: x, y, z = 32, 2, −28; *P-corrected*<0.03; Figure 4 a,b).

The amygdala is a complex nuclear structure. It is interconnected with several cortical areas and subcortical brain centers and subserves a variety of functional roles. However, a fundamental amygdalar function is to provide neutral stimuli with positive or negative values through association learning (e.g. [40]–[43]).

For a long time, studies involving the amygdala have mainly focused on negative stimulus conditioning. However, more recent studies support a role of the amygdala also for positive emotions, both in animals [43] and humans (e.g. [42]). This property puts the amygdala as a prime candidate in the storing of implicit emotional memories that can be subsequently accessed and used. In this light, the judged-as-beautiful stimuli could have been judged as such, not on the basis of their objective parameters, but because they were associated with memories charged with positive emotional values. The distinctiveness of each own experience would then partly explain the variance observed in the subjective rating of the observed images.

Finally, we compared judged-as-ugly versus judged-as-beautiful stimuli. As shown in Figure 4c, the only activated area was a region straddling the central sulcus (somatomotor cortices; *P*-corrected<0.05; see also Table 3a). Figure 4d shows signal change in this region, revealing a particularly strong effect of “ugly” versus “beautiful” images during the explicit aesthetic judgment condition. This selectivity was confirmed by the significant stimulus-by-condition interaction, as reported in Table 3.

**Table 3:** Brain activity reflecting main effect (a) and interaction (b) of judged-as-ugly *vs.* judged-as-beautiful images.

Brain structure	Sphere	Maxima x y z	Z	p. corr Cluster level
**a Main effect**				
Precentral gyrus	L	−36, −14, 60	4.68	0.0001
Postcentral gyrus	L	−38, −28, 52	4.34	
**b Interaction (stimulus by condition)**				
Precentral gyrus	L	−36, −12, 58	4.35	0.003
Postcentral gyrus	L	−40, −34, 56	3.88	
Inferior parietal lobule	L	−50, −26, 40	3.82	
	L	−52, −32, 52	3.34	

These data are in accord with previous findings by Kawabata and Zeki [6] showing that a negative evaluation of paintings (landscapes, abstract paintings, portraits, still life) determined the activation of the somatomotor region. There is also evidence from other studies that negative emotional stimuli may determine unilateral or bilateral activation in this region (e.g. fear, [44]; anger, [45], [46]).

The activation of the somatomotor region during aesthetic judgment seems rather surprising in the absence of actual movements. However, this activation may find an explanation if one also considers the activity pattern (*deactivation*) of the orbito-frontal cortex reported in [6] and also found in our work in a post-hoc analysis (see Supporting Information Text S1 and Figure S1). Although much attention has been drawn in recent years on the role of the orbito-frontal cortex in relation to positive rewards (for a review, see [47], [48]), there is also evidence coming from lesion studies that damage to orbitofrontal cortex causes a liberation of a variety of behaviors, ranging from extreme irritability, hot temper, antisocial behavior, to euphoria, locomotor hyperactivity and sexual disinhibition (e.g. [49]; for a review see [50]). If one admits that a decrease of activity in orbito-frontal cortex mimics, although to a different extent, the effect of a lesion one may account for the motor activation in response to ugly stimuli as a covert release of an appropriate motor behavior.

### Final considerations

The main question we addressed in the present study was whether there is an objective beauty, i.e., if objective parameters intrinsic to works of art are able to elicit a specific neural pattern underlying the sense of beauty in the observer. Our results gave a positive answer to this question. The presence of a specific parameter (the golden ratio) in the stimuli we presented determined brain activations different to those where this parameter was violated. The spark that changed the perception of a sculpture from “ugly” to beautiful appears to be the *joint* activation of specific populations of cortical neurons responding to the physical properties of the stimuli and of neurons located in the anterior insula.

Insula mediates emotion *feelings*. It would be too reductive, however, to think that the sense of beauty occurs because of the activation of this structure alone. Insula is also activated by non-artistic stimuli; however, the feeling that these stimuli produce in the observer differs *qualitatively* from that determined by artworks. Our view is that this specific quality–the sense of beauty-derives from a *joint* activity of neural cortical populations responsive to specific elementary or high order features present in works of art and neurons located in emotion controlling centers.

It has often been claimed that beauty, objectively determined, does not exist because of profound subjective differences in the evaluation of what is beautiful and what is not. Although individual biases are undeniable, it is also rather implausible to maintain that beauty has no biological substrate and is merely a conventional, experientially determined concept. As Gombrich [51] wrote, elements in a picture which determine aesthetical experience are “deeply involved in our biological heritage”, although we are unable to give a conscious explanation to them (see also [52]).

The results of our experiment concerning what we called *subjective* beauty are also relevant here. In the condition in which the viewers were asked to indicate explicitly which sculptures they liked, there was a strong increase in the activity of the amygdala, a structure that responds to incoming information laden with emotional value. Thus, instead of allowing their nervous centers to “resonate” in response to the observed stimuli (observation condition), when the viewers judged the stimuli according to their individual idiosyncratic criteria (explicit aesthetic judgment), that structure was activated that signals which stimuli had produced pleasant experiences in the past.

In conclusion, both objective and subjective factors intervene in determining our appreciation of an artwork. The history of art is replete with the constant tension between objective values and subjective judgments. This tension is deepened when artists discover new aesthetic parameters that may appeal for various reasons, be they related to our biological heritage, or simply to fashion or novelty. Still, the central question remains: when the fashion and novelty expire, could their work ever become a permanent patrimony of humankind without a resonance induced by some biologically inherent parameters?

## Supporting Information

Text S1 Preliminary behavioral study: description. Post hoc analysis: orbito-frontal cortex.
(0.03 MB DOC)

Figure S1 Deactivation pattern of judged-as-ugly sculpture images. Statistical parametric maps rendered onto the MNI brain template showing activity in the contrast “rest vs. judged-as-ugly stimuli” across conditions (O, AJ, PJ).
(3.07 MB TIF)
