# Supplementary material for: The Golden Beauty: Brain Response to Classical and Renaissance Sculptures
Source: PLoS One. 2007 Nov 21;2(11):e1201. doi: 10.1371/journal.pone.0001201 (PMC2065898; doi:10.1371/journal.pone.0001201)
Supplement: Text S1 — Preliminary behavioral study: description. Post hoc analysis: orbito-frontal cortex. (0.03 MB DOC) [file pone.0001201.s001.doc]

**Supporting Information**

**PRELIMINARY BEHAVIORAL STUDY**

Prior to MR scanning, we performed a behavioral study to select the images of sculptures to use in the brain imaging experiment.

**Participants** Twenty-five naïve healthy right-handed volunteers (12 males, 13 females; age range 22 – 32 years) took part in the preliminary behavioral study. They were educated undergraduate or graduate students, with no experience in art theory. After receiving an explanation of the experimental procedure, participants gave their written informed consent.

**Stimuli** Twenty-nine 2-dimentional images of sculptures representing human bodies were chosen from Classic and Renaissance art. All pictures met the criteria of canonical proportions defined by the ratio 1:1.618 between body parts (range ratio trunk:legs = 1:1.60 – 1:1.63). A second set of 58 stimuli was created from the canonical images by modifying graphically the ratio trunk-legs in two different ways. Half of the modified images were created according to a ‘long-trunk, short-legs’ modification (range = 1:1.45 - 1:1.59), whereas the remaining 29 images presented the opposite pattern of modification (range = 1:1.64 - 1:1.82).

The modifications were performed using a photo editing computer program (GIMP; version 2.2). The body-segments were scaled using a cubic interpolation method. The modifications of proportion were moderate in order to create plausible body structures that nonetheless violated the standard parameter of aesthetics. All the images were grey-scaled, equally sized (head-foot: 655px) and were presented in a frontal view on a black background.

**Task** Volunteers were presented with the images of sculptures on a computer screen and were asked to express an aesthetic judgment. Each trial began with a 1.5 s central fixation. Then the stimulus was presented centrally for 2 or 5 seconds (see below) and the volunteers were required to express an aesthetic preference on a scale ranging from 1 (very ugly) to 5 (very beautiful). Volunteers responded by pressing keys 1 to 5 on the computer keyboard. The maximum response time allowed was 7.5 seconds, but the next trial began as soon as the volunteer pressed a response key. Within the experimental session each of the 87 possible images (see above) was presented twice: once with a short presentation time (2 s) and once with a long presentation time (5 s). The stimuli were presented randomly, with presentation-times (short, long) and image-type (canonical, modified) intermixed within the same experimental session. The experimental session lasted for maximum 26 minutes.

**Stimulus selection** This test aimed at the selection of 30 experimental stimuli to be employed for the fMRI study (15 canonical and 15 modified images). A 2x2 repeated measures ANOVA with stimulus type (canonical and modified) and presentation-time (long and short) as independent factors was employed to test for any effects of stimulus type and presentation delay on aesthetic rating.

A cut-off point of 3 was then employed to assign positive ratings to the images, i.e. images that scored 3 to 5 were rated as beautiful (considering now only scores obtained in the 2 s delay – see Results). On this basis, for the fMRI protocol we selected the 15 original-modified pairs producing the greatest rating difference (i.e. the lowest scores for the modified and the highest scores for the original images).

**Results** Data obtained from a 2x2 repeated measures ANOVA revealed a main effect of stimulus type (canonical *vs*. modified; *F*(1,24) = 82.27, *P*<.0001), and no effect of stimulus delay (2 *vs*. 5 s; *P*>0.05). This allowed us to reliably select 2 s as stimulus presentation time during scanning. Violation of canonical proportions accounted for 77% of the variance in aesthetic rating (partial Eta^2^), supporting a strong effect of proportion on aesthetic appreciation.

The canonical / modified pairs which produced the greatest difference between mean ratings were then selected as the stimuli to be used in the fMRI experiment. Following this procedure, we selected a pool of stimuli composed of 15 canonical images (mean rating = 3.43; sd = 0.7) and 15 modified images (mean rating = 2.4; sd = 0.5).

**POST HOC ANALYSIS**

**Judged-as-ugly *vs*. judged-as-beautiful sculptures**

A post-hoc analysis was performed to better understand the activation pattern observed for the contrast “judged-as-ugly *vs*. judged-as-beautiful” images found during the subjective analysis of the data.

Firstly, we carried out a main effect analysis contrasting rest *vs*. judged-as-beautiful and judged-as-ugly images, modeled for each condition (observation, aesthetic judgment and proportion judgment, ANOVA). For this analysis, we used *P*-corrected = 0.05 at the cluster-level (cluster size estimated with a voxel-level threshold of *P*-uncorrected = 0.001; extent threshold = 10 voxels). Most importantly, to compare activation pattern in this study with that obtained in Kawabata and Zeki’s (2004), within the main effect analysis we carried out a small volume correction procedure (Worsley *et al*., 1996) to test for the effect of ‘judged-as-ugly’ images in orbito-frontal cortex (rest *vs*. ugly; across O/AJ/PJ conditions). Accordingly, the search volume was derived from Kawabata and Zeki (2004), centring a sphere at MNI coordinates x, y, z = -4, 38, -22, corresponding to Talairach coordinates x, y, z = -4, 36, -20; extent threshold = 10 voxels.

**Results** As shown in Supplementary Fig. 1, the main effect analysis contrasting the deactivation pattern of judged-as-ugly images revealed signal change in the medial frontal cortex bilaterally (-6, 36, -6; 8, 52, -6), the superior temporal sulcus bilaterally (62, -58, 20; -44, -62, 22), the left middle temporal gyrus (-44, -62, 22) and the precuneus bilaterally (-8, 58, 20; 10, -50, 38).

Additionally, the small volume procedure carried out to ascertain correspondence between the orbito-frontal area found by Kawabata and Zeki’s and our work supported significant overlap (-4, 38, 12; P = 0.01, corrected for small volume). This allowed us to interpret our results also in consideration of the findings obtained in Kawabata and Zeki’s work. The effect of judged-as-ugly stimuli was particularly strong during explicit aesthetic judgment, which is in line with the observation that the activity in the motor cortex during the contrast judged-as-ugly vs. judged-as-beautiful stimuli was mainly driven by a negative evaluation of the stimuli as expressed during AJ condition.

**Supplementary Figures Legend**

**Figure S1. Deactivation pattern of judged-as-ugly sculpture images**. Statistical parametric maps rendered onto the MNI brain template showing activity in the contrast “rest vs. judged-as-ugly stimuli” across conditions (O, AJ, PJ).
