# Supplementary figures and images for: The Golden Beauty: Brain Response to Classical and Renaissance Sculptures
Source: PLoS One. 2007 Nov 21;2(11):e1201. doi: 10.1371/journal.pone.0001201 (PMC2065898; doi:10.1371/journal.pone.0001201)

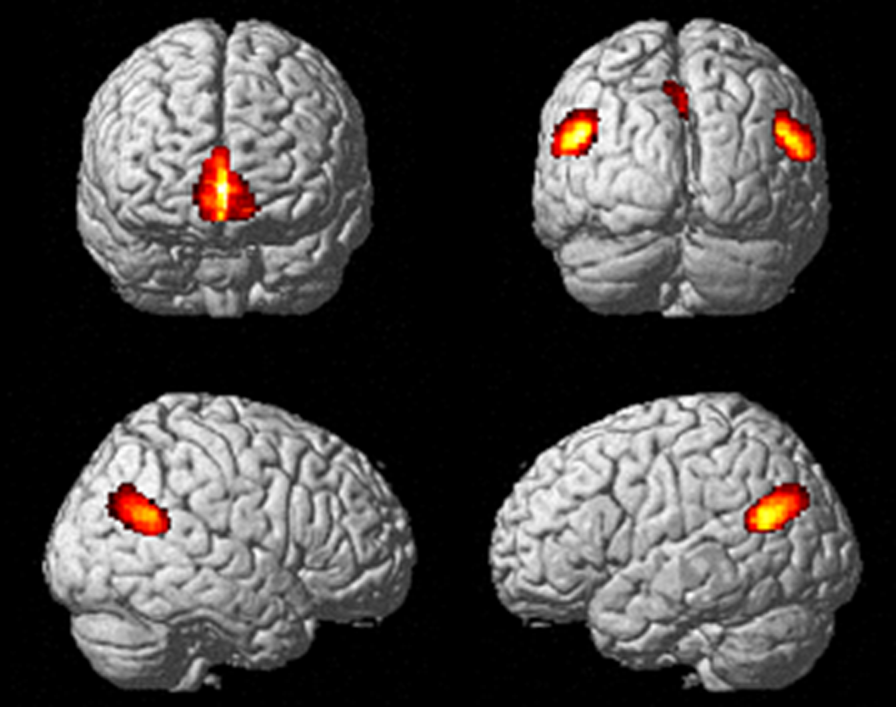

Supplement: Figure S1 — Deactivation pattern of judged-as-ugly sculpture images. Statistical parametric maps rendered onto the MNI brain template showing activity in the contrast “rest vs. judged-as-ugly stimuli” across conditions (O, AJ, PJ). (3.07 MB TIF) [file pone.0001201.s002.tif]
